# Hypertension, overweight/obesity, and diabetes among immigrants in the United States: an analysis of the 2010–2016 National Health Interview Survey

**DOI:** 10.1186/s12889-018-5683-3

**Published:** 2018-06-20

**Authors:** Yvonne Commodore-Mensah, Elizabeth Selvin, Jonathan Aboagye, Ruth-Alma Turkson-Ocran, Ximin Li, Cheryl Dennison Himmelfarb, Rexford S. Ahima, Lisa A. Cooper

**Affiliations:** 1 Department of Community-Public Health, Johns Hopkins University School of Nursing, 525 N. Wolfe Street, Room 419, Baltimore, MD 21205 USA; 2 Department of Epidemiology, Johns Hopkins Bloomberg School of Public Health, 2024 E. Monument Street, Suite 2-600, Baltimore, MD 21287 USA; 3 School of Medicine, Johns Hopkins University, 733 N Broadway, Baltimore, MD 21205 USA; 4 Johns Hopkins School of Nursing, 525 N. Wolfe Street, Baltimore, MD 21205 USA; 5 Department of Biostatistics, Johns Hopkins Bloomberg School of Public Health, 615 N Wolfe St, Baltimore, MD 21205 USA; 6 Department of Acute and Chronic Care, Johns Hopkins School of Nursing, 525 N. Wolfe Street, Baltimore, MD 21205 USA; 7 Division of Endocrinology, Diabetes and Metabolism, Johns Hopkins University School of Medicine, 1830 E. Monument Street, Suite 333, Baltimore, MD 21287 USA; 8 Department of Medicine, Johns Hopkins University School of Medicine, 2024 E. Monument Street, Suite #2-500, Baltimore, MD 21287 USA; 9 Department of Health, Behavior, and Society, Johns Hopkins Bloomberg School of Public Health, 2024 E. Monument St, Suite 2-500, Baltimore, MD 21287 USA

**Keywords:** Hypertension, Obesity, Diabetes, Immigrants, Ethnic minorities

## Abstract

**Background:**

Ethnic minority populations in the United States (US) are disproportionately affected by cardiovascular disease (CVD) risk factors, including hypertension, overweight/obesity, and diabetes. The size and diversity of ethnic minority immigrant populations in the US have increased substantially over the past three decades. However, most studies on immigrants in the US are limited to Asians and Hispanics; only a few have examined the prevalence of CVD risk factors across diverse immigrant populations. The prevalence of diagnosed hypertension, overweight/obesity, and diagnosed diabetes was examined and contrasted among a socioeconomically diverse sample of immigrants. It was hypothesized that considerable variability would exist in the prevalence of hypertension, overweight and diabetes.

**Methods:**

A cross-sectional analysis of the 2010–2016 National Health Interview Survey (NHIS) was conducted among 41,717 immigrants born in Europe, South America, Mexico/Central America/Caribbean, Russia, Africa, Middle East, Indian subcontinent, Asia and Southeast Asia. The outcomes were the prevalence of diagnosed hypertension, overweight/obesity, and diagnosed diabetes.

**Results:**

The highest multivariable adjusted prevalence of diagnosed hypertension was observed in Russian (24.2%) and Southeast Asian immigrants (23.5%). Immigrants from Mexico/Central America/Caribbean and the Indian subcontinent had the highest prevalence of overweight/obesity (71.5 and 73.4%, respectively) and diagnosed diabetes (9.6 and 10.1%, respectively). Compared to European immigrants, immigrants from Mexico/Central America/Caribbean and the Indian subcontinent respectively had higher prevalence of overweight/obesity (Prevalence Ratio (PR): 1.19[95% CI, 1.13–1.24]) and (PR: 1.22[95% CI, 1.14–1.29]), and diabetes (PR: 1.70[95% CI, 1.42–2.03]) and (PR: 1.78[95% CI, 1.36–2.32]). African immigrants and Middle Eastern immigrants had a higher prevalence of diabetes (PR: 1.41[95% CI, 1.01–1.96]) and PR: 1.57(95% CI: 1.09–2.25), respectively, than European immigrants —without a corresponding higher prevalence of overweight/obesity.

**Conclusions:**

Immigrants from Mexico/Central America/Caribbean and the Indian subcontinent bore the highest burden of overweight/obesity and diabetes while those from Southeast Asia and Russia bore the highest burden of hypertension.

## Background

Cardiovascular disease (CVD) is the leading cause of death in the United States (US). In 2015 one in three deaths was attributed to CVD, with approximately one death occurring every 40 s [1]. The prevalence of hypertension, overweight/obesity, and diabetes in US adults remains high and is estimated at 33, 69% and 12.4, respectively [1, 2]. In 2015, direct and indirect costs related to CVD and stroke were reported to be over $316.6 billion [1] and $245 billion for diabetes [3]. Ethnic minority populations in the US are disproportionately affected by hypertension, diabetes, and overweight/obesity among other chronic conditions, mirroring global trends [1].

The rising trend of these CVD risk factors is not unique to the US; this trend is also seen globally [4]. The global prevalence of diabetes has almost doubled from 4.7% in 1980 to 8.5% in 2014. [5] Across World Health Organization (WHO) regions, the prevalence of hypertension is highest in Africa, where it was 46%, and lowest in the Americas at 35%. [6] Approximately 17.5 million deaths per year—31% of the deaths reported worldwide—are attributed to CVD. [1]

In 2015 there were over 43.3 million immigrants in the US, comprising 13.5% of the population; [7] it is projected that this immigrant population would increase to 18% by 2065 [8]. The top five countries of origin of immigrants in 2015 were India, China, Mexico, Philippines and Canada [8]. Due to the rapidly changing demographics of immigrants in the US, it is increasingly more important to address the health status of immigrants—given the global burden of CVD risk factors.

Few studies have compared the prevalence of CVD risk factors among diverse immigrant populations in the US. Studies have shown that immigrants to the US typically have better health when they arrive from their home countries than the US-born population; however, this advantage is lost with increasing years of residence in the US. [9] This phenomenon is referred to as the “healthy immigrant effect” and is attributed to changes in the socioeconomic, physical, and cultural environment [10]. The purpose of this study was to examine and contrast the prevalence of diagnosed hypertension, overweight/obesity, and diagnosed diabetes among a diverse sample of US immigrants and establish whether there is a high burden of CVD risk factors among US immigrants.

## Methods

### Study population

Data from the National Health Interview Survey (NHIS), a population-based survey of civilian, non-institutionalized US adults ≥18 years conducted by the National Center for Health Statistics (NCHS) were analyzed. A full description of the methodology used is published elsewhere [11]. Briefly, the NHIS utilizes a 3-stage stratified cluster probability design with an oversampling of Blacks and Hispanics and includes approximately 45,000 households and about 110,000 persons annually [11]. Face-to-face interviews are administered by study staff and one randomly selected adult per household completes the Sample Adult Module to provide detailed information on health care services, behavior, and health status. To improve the reliability of the estimates, data for years 2010–2016 were pooled using guidelines established by the NCHS [11].

### Participants

All respondents who reported being foreign-born were considered immigrants; this definition includes naturalized citizens, legal permanent residents, undocumented immigrants, and those on visas including students and guest workers. Region of birth data were identified by the NHIS question, “Where were you born?” Within this variable there are 9 mutually exclusive regions: Mexico/Central America/Caribbean (Mexico, all countries in Central America and the Caribbean Island area, including Puerto Rico), South America, Europe, Russia, Africa, the Middle East, the Indian subcontinent (including India, Afghanistan, Bangladesh, Pakistan, Nepal, Pakistan, Sri Lanka, and others), Asia (including Asia, Asia Minor, China, Japan, North Korea, South Korea, and others) and Southeast Asia (Cambodia, Hong Kong, Indonesia, Laos, Malaysia, Myanmar, Vietnam, Philippines, Singapore, South Vietnam, Taiwan, Thailand, and others). Details on all countries included in each of the regions are published elsewhere. [11]

### Measurements

Hypertension was defined as a self-reported history of hypertension documented by a doctor or other healthcare professional in the past 12 months. Diabetes was also defined as self-reported history of diabetes. Body mass index (BMI) was calculated from self-reported height and weight. Overweight/obesity was defined as a BMI ≥25 kg/m^2^ in non-Asian populations and BMI ≥23 kg/m^2^ in immigrants born on the Indian subcontinent, in Asia, and in Southeast Asia, per WHO guidelines [12]. Covariates included age, sex, length of stay in the US, health insurance status, and income. Respondents were asked how long they had resided in the US; responses were dichotomized into < 10 and ≥ 10 years. Health insurance status was recoded as private, public, or no coverage. Poverty income ratio (PIR) was calculated and recoded by the NCHS as defined as the midpoint family income divided by the poverty level in dollars, as defined by the US Census Bureau for the corresponding survey year. Of note, the PIR was created and recoded by the NCHS.

### Statistical analysis

Sampling weights for the years 2010–2016 were included to account for the sampling design [13]. The chi-squared test was used to assess differences in categorical variables and analysis of variance test to test for differences in continuous variables between immigrants from the nine regions of birth. The age- and sex-adjusted hypertension, overweight/obesity and diabetes prevalence by region of birth were estimated by fitting generalized linear models using a Poisson distribution and a log link to obtain the respective predicted probabilities. Multivariable models for each outcome were fitted by adjusting for sociodemographic characteristics and healthcare access. Age, poverty status, education, length of US residence and doctor’s visit in past 12 months were adjusted for. Stata’s margins command [14] was used to estimate and interpret the adjusted predictions and marginal effects for all the outcomes. Predictive margins are an appealing method of direct standardization because the predicted values from the Poisson regression models can be averaged over the covariate distribution of the population [15]. To determine whether the associations varied by sex, effect measure modification of sex was tested by creating an interaction term of the region of birth and sex for the three outcomes. The interactions terms were all significant with *p* < 0.0001 so the results were stratified by sex. Statistical analyses were performed with Stata® version 14.0. Europe was used as the reference group in the comparative analyses because, in health disparities research, ethnic minorities in the US are compared to Whites who are primarily of European-descent and size of the European immigrant population was large enough to allow for meaningful comparisons to the other groups.

## Results

### Sociodemographic characteristics of the study population

A total of 41,717 immigrants were included in this study. When weighted to the US population, these participants represented 15,688,540 immigrants in the US. The sample consisted of immigrants from Europe (12.5%), South America (6.6%), Mexico/Central America/Caribbean (47%), Russia (2.7%), Africa (5%), Middle East (3%), Indian subcontinent (6.5%), Asia (7.5%), and Southeast Asia (9%). The mean age ± SE of all immigrants was 46.5 (0.18) years. European immigrants were the oldest (53.5 years); immigrants from Africa and the Indian subcontinent were the youngest (41 years). There were significant differences in all sociodemographic characteristics examined across the nine immigrant groups (Table 1).

**Table 1:** Sociodemographic characteristics, by Region of Birth: 2010–2016 NHIS, *N* = 41,717

Mean (SE), *n* (%)	Europe (*N* = 3668)	South America (*N* = 2650)	Mexico/C. Amer/Carib (*N* = 21,870)	Russia (*N* = 688)	Africa (*N* = 1712)	Middle East (*N* = 870)	Indian sub. (2593)	Asia (*N* = 3214)	S.E. Asia (*N* = 4297)
Total immigrant population,	12.5 (0.3)	6.6 (0.2)	47 (0.6)	2.7 (0.2)	4.7 (0.2)	3.3 (0.2)	6.5 (0.2)	7.5 (0.3)	9.3 (0.2)
Age, yrs.^*^	53.5 (0.4)	47.2 (0.4)	45.6 (0.2)	47.6 (1.1)	41 (0.4)	44.7 (0.9)	40.7(0.5)	46.3 (0.6)	48.8 (0.3)
Male ^*^	43.1 (0.9)	44.7 (1.1)	47.2 (0.4)	44.1 (2.4)	54.4 (1.4)	55.7 (2.1)	54.7 (1.2)	42 (1.1)	42 (0.9)
Education ^*^									
≤ High School Degree	32.1 (1.1)	38.1 (1.3)	71.1 (0.5)	19.4 (1.9)	26.6 (1.3)	25.4 (1.9)	13.9 (0.9)	25.3 (1.3)	28.9 (9.6)
> High School & < College	28.7 (0.9)	27.4 (0.9)	17.3 (0.3)	24.9 (1.8)	29.7 (1.2)	23.9 (2.1)	8.5 (0.7)	20.3 (1.0)	24.4 (0.7)
≥ College	39.2 (1.2)	34.4 (1.2)	11.5 (0.3)	55.8 (2.1)	43.6 (1.4)	50.6 (2.5)	77.6 (1.2)	54.5 (1.4)	46.7 (1.0)
Poverty status*									
Poor	8.5 (0.7)	14.0 (0.8)	25.2 (0.4)	18.7 (2.3)	19.9 (1.3)	20.5 (1.9)	10.8 (0.8)	17.8 (1.2)	11.7 (0.7)
Near Poor	13.8 (0.6)	20.9 (1.0)	26.3 (0.4)	14.1 (2.5)	19.5 (1.1)	16.7 (1.5)	10.1 (0.7)	13.2 (0.7)	14.1 (0.6)
> Near Poor	77.8 (0.9)	65 (1.1)	48.5 (0.5)	67.2 (2.9)	60.5 (1.5)	62.8 (2.3)	79.0 (1.2)	69.0 (1.4)	74.2 (0.9)
≥ 10 yrs. US residence ^*^	88.5 (0.7)	78.9 (1.0)	83.7 (0.4)	77.6 (1.6)	63.4 (1.4)	63.1 (2.5)	55.2 (1.8)	70.4 (1.3)	81.7 (0.8)
Employed^*^	54.9 (1.0)	64.1 (1.1)	60.7 (0.5)	60.7 (2.3)	68.9 (1.3)	48.4 (1.9)	69.1 (1.0)	54.6 (1.3)	63.2 (0.9)
Health Insurance(Yes) ^*^	89.4 (0.6)	75.2 (1.0)	61.1 (0.7)	84.3 (1.6)	75.3 (1.2)	85.7 (1.3)	90.5 (0.6)	86.8 (0.8)	87.9 (0.6)
Seen doctor in past year(Yes)*	72.3 (0.9)	65.8 (1.0)	55.6 (0.5)	67 (2.0)	59.8 (1.4)	62.1 (2.1)	63.1 (1.2)	60.6 (1.1)	65.2 (0.9)

^*^- *p* < 0.001, Mexico/C. Amer/Carib = Mexico/Central America/Caribbean, Indian sub = Indian subcontinent, S.E. Asia = South East Asia

### Adjusted prevalence and prevalence ratios of hypertension, overweight/obesity, and diabetes by region of birth

**Fig. 1:**
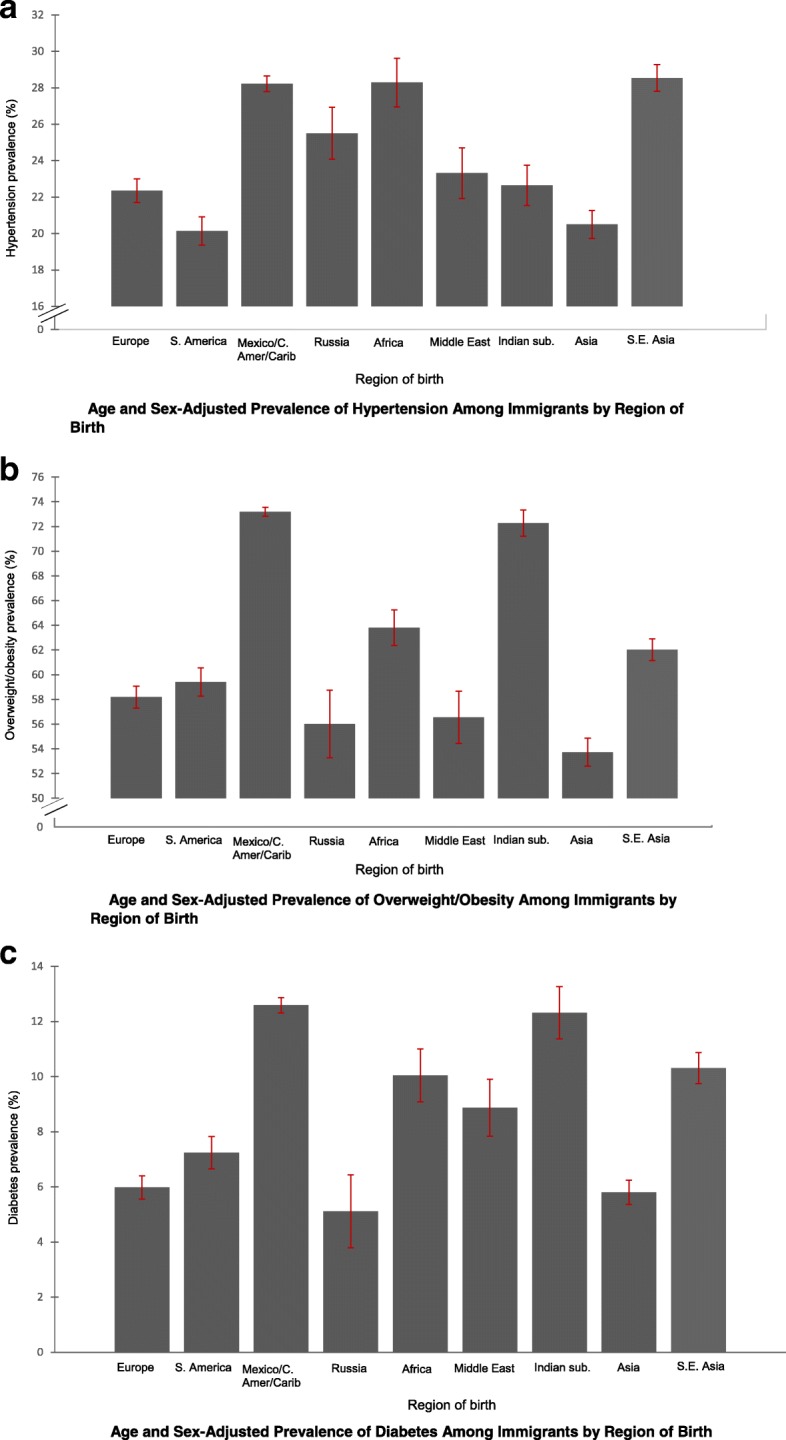
Age and Sex-Adjusted Prevalence of (**a**) Hypertension (**b**) Overweight/Obesity (**c**) Diabetes among Immigrants in the 2010–2016 National Health Interview Survey

The age- and sex-adjusted prevalence of hypertension, overweight/obesity, and diabetes are presented in Fig. 1a, b and c respectively. The absolute adjusted prevalence and adjusted prevalence ratios of the risk factors are presented in Tables 2 and 3 respectively and stratified by sex.

**Table 2:** Adjusted Prevalence of Hypertension, Overweight/Obesity, Diabetes among US Immigrants:2010–2016 NHIS

	Hypertension %(95%CI)			Overweight/Obesity %(95%CI)			Diabetes%(95%CI)		
Region of birth	Total (*N* = 41,717)	Males (*N* = 18,804)	Females (*N* = 22,913)	Total (*N* = 41,717)	Males (*N* = 18,804)	Females (*N* = 22,913)	Total (*N* = 41,717)	Males (*N* = 18,804)	Females (*N* = 22,913)
Europe	22.4 (21.1–23.7)	22.3 (20.5–24.2)	22.7 (21.0–24.4)	58.6 (56.8–60.4)	65.6 (62.9–68.2)	52.5 (50.0–55.0)	6.3 (5.4–7.2)	6.6 (5.3–7.8)	6.2 (5.0–7.4)
South America	20.0 (18.5–21.5)	18.1 (16.0–20.3)	21.7 (19.4–23.9)	59.4 (57.2–61.7)	68.4 (65.2–71.6)	51.7 (48.6–54.8)	7.3 (6.2–8.5)	6.3 (4.6–7.9)	8.1 (6.5–9.8)
Mexico/C. Amer/ Carib	27.7 (26.9–28.6)	24.9 (23.7–26.1)	30.3 (29.3–31.4)	70.7 (69.9–71.5)	75.2 (74.1–76.4)	66.7 (65.5–67.8)	11.6 (11.1–12.2)	10.5 (9.7–11.2)	12.6 (11.8–13.4)
Russia	26.0 (23.1–28.8)	27.8 (23.6–31.9)	24.4 (20.8–28.1)	57.0 (51.2–62.8)	65.5 (59.1–71.9)	49.7 (41.6–57.8)	5.2 (3.1–7.3)	6.5 (3.3–9.8)	4.1 (1.1–7.0)
Africa	28.8 (26.1–31.6)	27.7 (23.9–31.5)	29.3 (25.6–33.0)	65.5 (62.6–68.4)	65.5 (61.1–69.8)	67.2 (62.7–71.7)	10.8 (8.8–12.9)	12.0 (9.1-15.0)	9.0 (6.4–11.6)
Middle East	23.2 (20.6–25.9)	25.9 (22.0–29.8)	19.9 (16.6–23.3)	59.1 (54.9–63.2)	63.9 (58.6–69.2)	54.5 (48.1–60.9)	8.8 (6.7–10.8)	12.2 (9.1–15.4)	5.2 (2.7–7.7)
Indian sub.	23.3 (21.1–25.5)	21.8 (18.9–24.6)	24.3 (20.9–27.7)	77.6 (75.2–80.1)	80.4 (77.3–83.5)	76.0 (72.1–79.8)	14.3 (12.0–16.5)	16.3 (13.2–19.3)	11.4 (8.6–14.3)
Asia	21.1(19.6–22.7)	22.5 (20.1–25.0)	20.2 (18.4–22.0)	55.2 (52.9–57.5)	68.9 (65.8–72.0)	43.6 (40.4–46.7)	6.1 (5.2–7.0)	6.1 (4.6–7.7)	6.2 (5.0–7.3)
S.E. Asia	29.1(27.6–30.6)	27.3 (25.1–29.5)	30.9 (28.8–32.9)	63.3 (61.5–65.1)	73.2 (71.0–75.4)	55.3 (52.7–57.8)	11.1 (9.9–12.3)	10.9 (9.1–12.6)	11.3 (9.7–12.8)

CI-Confidence Interval; Prevalence adjusted for age, poverty status, education, duration of US residence, doctor’s visit in past 12 months; Mexico/C. Amer/Carib-Mexico/Central America/Caribbean; Indian sub-Indian subcontinent; S.E. Asia- South East Asia

**Table 3:** Adjusted Prevalence Ratios (PR) of Hypertension, Overweight/Obesity, Diabetes among US Immigrants, 2010–2016 NHIS

	Hypertension Prevalence Ratio (95% CI)			Overweight/Obesity Prevalence Ratio (95% CI)			Diabetes Prevalence Ratio (95% CI)		
	Total (*N* = 41,717)	Males (*n* = 18,804)	Females (*n* = 22,913)	Total (*N* = 41,717)	Males (*n* = 18,804)	Females (*n* = 22,913)	Total (*N* = 41,717)	Males (*n* = 18,804)	Females (*n* = 22,913)
Europe	*Reference*	*Reference*	*Reference*	*Reference*	*Reference*	*Reference*	*Reference*	*Reference*	*Reference*
South America	**0.89 (0.82–0.98)**	**0.81 (0.70–0.94)**	0.95 (0.84–1.08)	1.01 (0.97–1.07)	1.04 (0.98–1.11)	0.98 (0.91–1.06)	1.16 (0.94–1.43)	0.96 (0.70–1.30)	1.32 (0.99–1.75)
Mexico/C. Amer/ Carib	**1.24 (1.17–1.32)**	**1.11 (1.01–1.22)**	**1.34 (1.23–1.45)**	**1.21 (1.17–1.25)**	**1.15 (1.10–1.20)**	**1.27 (1.21–1.34)**	**1.84 (1.60–2.13)**	**1.60 (1.31–1.94)**	**2.04 (1.66–2.50)**
Russia	**1.16 (1.03–1.31)**	**1.24 (1.04–1.48)**	1.08 (0.92–1.25)	0.97 (0.88–1.08)	1.00 (0.90–1.11)	0.95 (0.80–1.12)	0.82 (0.55–1.24)	1.00 (0.59–1.70)	0.66 (0.31–1.38)
Africa	**1.29 (1.15–1.44)**	**1.24 (1.06–1.45)**	**1.29 (1.12–1.49)**	**1.12 (1.06–1.18)**	1.00 (0.92–1.08)	**1.28 (1.18–1.39)**	**1.71 (1.38–2.13)**	**1.84 (1.38–2.46)**	**1.46 (1.05–2.03)**
Middle East	1.04 (0.92–1.17)	1.16 (0.97–1.38)	0.88 (0.74–1.04)	1.01 (0.94–1.09)	0.97 (0.89–1.07)	1.04 (0.92–1.17)	**1.39 (1.07–1.82)**	**1.87 (1.35–2.58)**	0.84 (0.50–1.43)
Indian sub.	1.04 (0.94–1.15)	0.97 (0.84–1.13)	1.07 (0.92–1.26)	**1.33 (1.27–1.38**)	**1.23 (1.16–1.30)**	**1.45 (1.35–1.54)**	**2.26 (1.83–2.79)**	**2.49 (1.91–3.24)**	**1.85 (1.36–2.50)**
Asia	0.94 (0.87–1.03)	1.01 (0.89–1.14)	**0.89 (0.80–0.99)**	**0.94 (0.89–0.99)**	1.05 (0.99–1.11)	**0.83 (0.76–0.90)**	0.97 (0.80–1.17)	0.93 (0.69–1.26)	1.00 (0.77–1.29)
S.E. Asia	**1.30 (1.21–1.40)**	**1.22 (1.09–1.37)**	**1.36 (1.24–1.49)**	**1.08 (1.04–1.12)**	**1.12 (1.06–1.17)**	1.05 (0.99–1.12)	**1.76 (1.49–2.08)**	**1.66 (1.32–2.08)**	**1.83 (1.45–2.29)**

CI-Confidence Interval; Prevalence adjusted for age, poverty status, education, duration of US residence, doctor’s visit in past 12 months; Mexico/C. Amer/Carib = Mexico/Central America/Caribbean; Indian sub = Indian subcontinent; S.E. Asia; bold- *p* < 0.05

The lowest prevalence of hypertension was observed in South American immigrants (20%) and the highest in Southeast Asian immigrants (29.1%). Immigrants from Southeast Asia, Africa and Mexico/Central America/Caribbean had higher, while those from South America had lower prevalence of hypertension than European immigrants. Female Asian immigrants reported hypertension diagnosis at lower rates than did female European immigrants. Male Russian immigrants had higher prevalence of hypertension than male European immigrants.

The lowest adjusted-overweight/obesity prevalence was in Asian immigrants (55.2%) and the highest was in immigrants from the Indian subcontinent (77.6%). Immigrants from Mexico/Central America/Caribbean and the Indian subcontinent had higher prevalence of overweight/obese than European immigrants. The adjusted prevalence of overweight/obesity among male Indian subcontinent immigrants was 80.4%. Female Asian immigrants had lower prevalence of overweight/obese than female European immigrants.

The lowest adjusted diabetes prevalence was observed in Russian immigrants (5.2%); the highest was observed in immigrants from the Indian subcontinent. (14.3%). Males from the Indian subcontinent had a remarkably high prevalence of diabetes (16.3%). Immigrants from Mexico/Central America/Caribbean, Africa, Middle East, Southeast Asia and the Indian subcontinent had higher prevalence of diabetes than those from Europe. It is noteworthy that the immigrant groups with the highest prevalence of overweight/obesity (Mexico/Central America/Caribbean and Indian subcontinent) also had the highest prevalence of diabetes.

## Discussion

This study was conducted to examine the prevalence of hypertension, overweight/obesity, and diabetes among immigrants who participated in the 2010–2016 NHIS. Considerable heterogeneity was observed in the prevalence of these risk factors; however, some groups bore the highest burden of risk. Understanding the distribution of CVD risk factors among immigrants is an essential area in epidemiology because of current trends in migration flows and effects on changes in the environment, social context, and health care access on cardiovascular health. The healthy immigrant effect, in the context of cardiovascular health, may differ for particular immigrant groups and the apparent advantage may be short-lived.

Per estimates derived from the 2013 Behavior Risk Factor Surveillance Survey, the prevalence of diagnosed hypertension is 32.5% [16], with ethnic minorities disproportionately affected. The overall prevalence of diagnosed hypertension in the 2013 NHIS was 24% [17]; Asian and White adults reported lower rates of diagnosed hypertension than Blacks. The age and sex-adjusted prevalence of diagnosed hypertension (28%) for immigrants from Mexico/Central America/Caribbean in our study is higher than previous estimates (20%) for this group in the 2012 NHIS [17] and the 26% estimate in the Hispanic Community Health Study/Study of Latinos [18]. The adjusted prevalence of hypertension is slightly lower than the 30% prevalence reported for this group in the National Health and Nutrition Examination Survey (NHANES) [1]. Since there is lower awareness of hypertension among Hispanics than among non-Hispanic Whites [18], hypertension prevalence may be underestimated in this study.

The highest hypertension prevalence was expected among African immigrants because their African-American counterparts have among the highest hypertension prevalence globally [19] and because hypertension prevalence has increased considerably in Africa [20]. The age- and sex-adjusted prevalence of hypertension (28%) observed in African immigrants in our study is substantially lower than the age-adjusted prevalence of diagnosed hypertension in African Americans (33%) in the 2013 NHIS [17]. However, since hypertension prevalence rises with increased length of stay in the US [9], this advantage in African immigrants may be lost with increased length of stay. The lower hypertension prevalence among African immigrants also supports previous calls to disaggregate data on African-descent populations in the US [21, 22] to identify contributors of disparities and protective factors associated with hypertension in Blacks.

Southeast Asian immigrants—which includes those born in Vietnam, Thailand, and the Philippines—also had one of the highest prevalence of hypertension (29%) and were more likely to have hypertension than European immigrants. Although this group spans disparate geographical regions, our results are consistent with prior studies which have shown that Southeast Asian immigrants are more likely to report hypertension than Whites. [23] In Filipino immigrants (*N* = 1028), Ursua and colleagues identified 53% of participants as hypertensive, a prevalence substantially higher than estimates obtained for Blacks (37%) and Hispanics (32%) [24] in that study. This observation may be explained by the limited availability of fresh vegetables, overconsumption of processed foods and increased sedentary behaviors among Southeast Asians [25]. The diversity of the Southeast Asian immigrant population may present a challenge for healthcare providers serving this population. However, risk reduction strategies that are ethnically-tailored, scientifically-valid and multifaceted hold promise for successfully reducing the burden of hypertension in Southeast Asian immigrants [26].

The age- and sex-adjusted prevalence of overweight/obesity was over 72% in Mexico/Central America/Caribbean and Indian subcontinent immigrants. It is striking that the adjusted prevalence of overweight/obesity among male Indian subcontinent immigrants was 80%. The adjusted prevalence among immigrants from the Indian subcontinent increased markedly (from 50 to 80%) when the Asian-specific BMI cut-offs were used in this study, highlighting the importance of using recommended BMI cut-offs among Asians. [12] More than 71% of adults in Mexico are currently overweight [27] which is almost identical to the prevalence observed in Mexico/Central America/Caribbean immigrants in our study. The prevalence of overweight/obesity has tripled in Mexico over the past 30 years; it is projected that by 2050 only 12% of males and 9% of females will have healthy weight [27]. Interventions to halt the rising tide of obesity prevalence in the Mexico/Central America/Caribbean region may prevent adverse cardiovascular outcomes for those who choose to migrate to the US.

The lowest adjusted prevalence of overweight/obesity was among Asian immigrants. Although Asian immigrants in the US are diverse, it is well-known that this population develops diabetes at lower BMIs than among Whites [28]. In our study, Southeast Asian immigrants were distinct and had a significantly higher prevalence of risk factors than the other Asian immigrants. This finding suggests that migration-related changes in health behaviors may be more pronounced among Southeast Asian immigrants and warrants culturally-tailored public health strategies. Since the majority (77%) of Southeast Asian immigrants in the US are from the Philippines and Vietnam [29], targeting these groups may have the highest impact.

The highest adjusted prevalence of diabetes was observed among Indian subcontinent and Mexico/Central America/Caribbean immigrants who also had a corresponding high overweight/obesity prevalence. These results are consistent with Oza-Frank and Narayan’s findings using the 1997–2005 NHIS [30]. The high prevalence of overweight/obesity and diabetes among immigrants from the Indian subcontinent was surprising. Of the 1.38 million immigrants who moved to the US in 2015, India was the leading country of origin with 179,800 arriving in 2015 [7]. As immigrants from the Indian subcontinent become integrated into the US society, clinical and public health strategies to reduce CVD risk must consider the susceptibility of this group to insulin resistance and hyperinsulinemia with greater visceral adiposity than Whites despite lower BMI [31].

The high prevalence of overweight/obesity and diabetes observed in Mexican/Central American/Caribbean immigrants is consistent with prior research. Previous studies have shown that Hispanics (broadly defined) have higher diabetes prevalence than Whites or Asian Americans [32]. The high diabetes prevalence in Mexico/Central America/Caribbean immigrants reflects the growing epidemic of diabetes in Mexico where diabetes prevalence is the leading cause of death and diabetes control is abysmal at 5.3% [33]. As Mexicans migrate to the US, poor health behaviors such as excessive carbohydrate intake and physical inactivity—and poor glycemic control may be exacerbated if they reside in obesogenic environments and are improperly integrated into the US healthcare system.

Importantly, this study has demonstrated that South American and Mexico/Central America/ Caribbean immigrants are distinct groups with different risk profiles because South Americans and Mexicans had the lowest and highest prevalence of the risk factors examined, respectively. In the Hispanic Community Health Study/Study of Latinos [34], 17% were diabetic. However, South Americans had the lowest prevalence (10%) while Mexicans had the highest (18%). From a clinical perspective, the presumption that “Hispanics/Latinos” are homogenous can lead to incorrect inferences that mask significant and actionable health information. The variation in the risk factor prevalence across ethnic subgroups could be explained by differences in diet (e.g., sodium intake), acculturation, or differences in pre-migration contexts of immigrants. A healthcare provider who simply identifies a patient as “Hispanic/Latino” without probing the specific cultural background, diet and perceptions of hypertension and diabetes misses a critical opportunity to provide culturally-sensitive and patient-centered care.

The strengths of this study include the use of a large representative sample of civilian, non-institutionalized US immigrants and pooling of seven years of data to increase statistical power and permit meaningful comparisons across nine regions of birth. The Asian-specific cut-offs for BMI were used, as recommended by the WHO [12]. This study has also provided critical data on other immigrant groups (Africans, Middle Eastern) who are not well-characterized in health disparities research. However, some limitations should be discussed. First, hypertension, diabetes, height, and weight were all self-reported, which may have resulted in an underestimation of our prevalence estimates. Second, since this study was a cross-sectional study, no inferences can be made regarding whether these risk factors were acquired before or after migration to the US. Third, the sample sizes of immigrants from Russia, Africa, and the Middle East are relatively smaller than the other groups; hence, our estimates may be less precise. Finally, data on Canadian immigrants are not publically reported in the NHIS although this group makes up on the top five immigrant groups in the US [8].

## Conclusions

The results of this study demonstrate considerable heterogeneity in the prevalence of diagnosed hypertension, overweight/obesity, and diagnosed diabetes among US immigrants. Immigrants from the Indian subcontinent and Mexico/Central America/Caribbean had the highest overweight/obesity and diabetes prevalence while those from Russia and Southeast Asia had the highest hypertension prevalence. Disaggregating data by country of origin will permit a better understanding of the various biologic and sociocultural factors that contribute to these CVD risk factors and ultimately the design, delivery and support of culturally tailored interventions. Additionally, cohort studies of diverse immigrant groups may improve our understanding of cardiovascular health trajectories, provide etiological insights into the development of CVD and provide stronger evidence of the healthy immigrant effect.

## Abbreviations

BMI: Body Mass Index
CVD: Cardiovascular Disease
NCHS: National Center for Health Statistics
NHANES: National Health and Nutrition Examination Survey
NHIS: National Health Interview Survey
P.R: Prevalence Ratio
US: United States
